# Dietary nitrate improves vascular function in patients with hypercholesterolemia: a randomized, double-blind, placebo-controlled study^1^,^2^,^3^

**DOI:** 10.3945/ajcn.115.116244

**Published:** 2015-11-25

**Authors:** Shanti Velmurugan, Jasmine Ming Gan, Krishnaraj S Rathod, Rayomand S Khambata, Suborno M Ghosh, Amy Hartley, Sven Van Eijl, Virag Sagi-Kiss, Tahseen A Chowdhury, Mike Curtis, Gunter GC Kuhnle, William G Wade, Amrita Ahluwalia

**Affiliations:** 4 William Harvey Research Institute, National Institute for Health Research Cardiovascular Biomedical Research Unit,; 5 Institute of Dentistry, and; 6 Blizard Institute, Barts & The London School of Medicine & Dentistry, Queen Mary University of London, London, United Kingdom;; 7 Department of Food and Nutritional Sciences, University of Reading, Reading, United Kingdom; and; 8 Barts National Health Service Trust, Department of Diabetes and Metabolic Medicine, The Royal London Hospital, London, United Kingdom

**Keywords:** endothelium, microbiome, nitric oxide, vascular, vegetable

## Abstract

**Background:** The beneficial cardiovascular effects of vegetables may be underpinned by their high inorganic nitrate content.

**Objective:** We sought to examine the effects of a 6-wk once-daily intake of dietary nitrate (nitrate-rich beetroot juice) compared with placebo intake (nitrate-depleted beetroot juice) on vascular and platelet function in untreated hypercholesterolemics.

**Design:** A total of 69 subjects were recruited in this randomized, double-blind, placebo-controlled parallel study. The primary endpoint was the change in vascular function determined with the use of ultrasound flow-mediated dilatation (FMD).

**Results:** Baseline characteristics were similar between the groups, with primary outcome data available for 67 patients. Dietary nitrate resulted in an absolute increase in the FMD response of 1.1% (an ∼24% improvement from baseline) with a worsening of 0.3% in the placebo group (*P* < 0.001). A small improvement in the aortic pulse wave velocity (i.e., a decrease of 0.22 m/s; 95% CI: −0.4, −0.3 m/s) was evident in the nitrate group, showing a trend (*P* = 0.06) to improvement in comparison with the placebo group. Dietary nitrate also caused a small but significant reduction (7.6%) in platelet-monocyte aggregates compared with an increase of 10.1% in the placebo group (*P* = 0.004), with statistically significant reductions in stimulated (ex vivo) P-selectin expression compared with the placebo group (*P* < 0.05) but no significant changes in unstimulated expression. No adverse effects of dietary nitrate were detected. The composition of the salivary microbiome was altered after the nitrate treatment but not after the placebo treatment (*P* < 0.01). The proportions of 78 bacterial taxa were different after the nitrate treatment; of those taxa present, 2 taxa were responsible for >1% of this change, with the proportions of *Rothia mucilaginosa* trending to increase and *Neisseria flavescens* (*P* < 0.01) increased after nitrate treatment relative to after placebo treatment.

**Conclusions:** Sustained dietary nitrate ingestion improves vascular function in hypercholesterolemic patients. These changes are associated with alterations in the oral microbiome and, in particular, nitrate-reducing genera. Our findings provide additional support for the assessment of the potential of dietary nitrate as a preventative strategy against atherogenesis in larger cohorts. This trial was registered at clinicaltrials.gov as NCT01493752.

## INTRODUCTION

Currently, there is a relative paucity in broadly acceptable effective primary prevention options for cardiovascular disease (CVD)^9^ (1, 2). In this regard, a focus on identifying strategies that target major but modifiable risk factors, particularly the diet, that might also operate as preventative strategies to avoid pharmacotherapeutics is clearly of value.

Vascular dysfunction is thought to play a major role in the progression of CVD, particularly in atherosclerosis (3). This change occurs at the very earliest stages of CVD development, preceding any evidence of disease (4), and is associated with most risk factors including hypercholesterolemia (5, 6). Indeed, measures of vascular function, particularly brachial artery flow-mediated dilatation (FMD) and aortic pulse wave velocity (aPWV), have been proposed to inform not only on the extent of atherosclerotic disease (4, 7, 8) but also of future risk (9, 10).

Mechanistically, vascular dysfunction is characterized by a reduced bioavailability of the endothelium-derived vasoprotective molecule NO. NO is a vasodilator with associated antiplatelet, anti-inflammatory, and antiproliferative effects that underlie its critical role in sustaining cardiovascular health (11). The dysfunction of the conventional l-arginine/NO–synthase pathway and enhanced scavenging of NO underlie the reduced bioavailability in individuals at risk of CVD including those with hypercholesterolemia (12), which is a phenomenon reflected by impaired FMD responses. Thus, approaches that might restore this lost NO have obvious therapeutic potential.

A dietary approach to provide the sustained restoration of nitric oxide concentrations on the basis of the delivery of inorganic nitrate has been identified. Inorganic nitrate is converted to nitrite by nitrate-reductase expressing bacteria, which naturally reside in the oral cavity. Once salivary nitrite is swallowed, some of it enters the systemic circulation, where it undergoes a subsequent chemical reduction to NO, which is a reaction that is facilitated by mammalian nitrite reductases (13). Previous evidence has shown that, although a single, acute dietary nitrate load acutely improves aPWV, it has no effect on endothelial function per se in healthy volunteers (14). However, a single dose of inorganic nitrate protects against a transient vascular dysfunction that is expressed as a decreased FMD, induced experimentally by an ischemia and reperfusion insult (15), as well as decreasing platelet sensitivity to activating stimuli (16). In addition, more recently in a study in which blood pressure (BP) was the primary outcome measure, sustained dietary nitrate ingestion in addition to lowering BP also improved vascular function in hypertensive patients, reflected by increased FMD responses (17). Thus, in a prospective trial, we sought to determine whether dietary nitrate as a method of NO delivery might provide an improvement in vascular function in a cohort of otherwise healthy hypercholesterolemics after a once-daily 6-wk dietary nitrate intervention in the form of nitrate-rich beetroot juice. In addition, we also assessed the possible effect on platelet function as a secondary outcome measure.

## METHODS

### Study design and subjects

This randomized, double-blind, placebo-controlled parallel trial was approved by the National Research Ethics Service Committee London-Stanmore (study 11/LO/0715) and complied with the Declaration of Helsinki. The trial was registered at clinicaltrials.gov as NCT01493752.

Subjects were recruited between September 2011 and May 2013 from advertisements within primary care facilities and from a dedicated cholesterol clinic within The Royal London Hospital. Sixty-nine nonsmoking, nondiabetic, otherwise healthy hypercholesterolemic men and women aged 18–80 y with BMI (in kg/m^2^) from 18.5 to 40 were recruited. Inclusion criteria included a total serum cholesterol concentration >6.0 mmol/L or any elevation of LDL cholesterol or triglycerides with a QRISK 2 (cardiovascular disease risk calculator; National Health Service) score >15%. All participants were expected to be free from any use of statins or other cholesterol-lowering medication for ≥2 mo before screening. All participants provided written informed consent before their inclusion in the study.

After successful screening, participants were randomly assigned 1:1 to receive daily either 250 mL naturally nitrate-rich beetroot juice or placebo nitrate-depleted beetroot juice (James Whites Drinks). The placebo juice was generated from the same batch of nitrate-replete juice by using a standard anion exchange resin as described previously (18). Visually, there was no detectable difference between the juices. Previous spectral, ion-concentration, sugar, and ascorbate content analysis and taste testing has confirmed no differences in color and constituents between the two types of juice (18, 19). All study personnel were blind to the treatment allocation until the study had been completed and all analyses were performed. The random assignment was generated via an online randomization program, and the packing of juice was undertaken by an individual who was not involved in patient contact or the sample collection and analysis. The code for the random assignment was only revealed once all data had been collected and computed. The random assignment was conducted in 2 blocks to enable a balance between the groups at the midpoint of recruitment.

**FIGURE 1:**
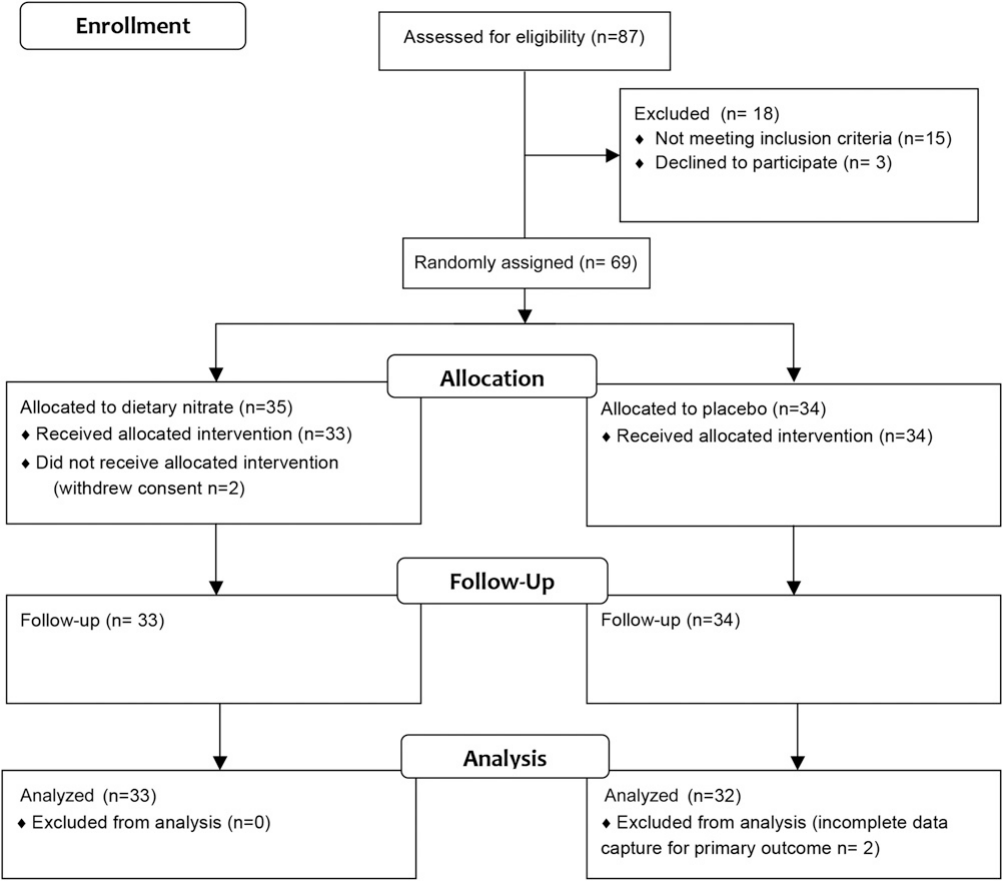
CONSORT flowchart of study. CONSORT, Consolidated Standards of Reporting Trials.

Participants were invited to attend their first study visit ≤2 wk of screening and were expected to withhold medications on the morning of study visits with any last medications consumed ≥12 h before study visits in the case of antihypertensives and 24 h before study visits in the case of aspirin. Participants were expected to fast 12 h before study visits having consumed a low-nitrate diet and having refrained from any strenuous exercise 24 h before study visits. Of 69 recruited subjects, 2 individuals withdrew consent at the time of the first visit (**Figure 1**). A single patient was taking 75 mg aspirin/d, and 14 patients were hypertensive and taking medications.

During each study visit, the FMD of the brachial artery, a pulse wave analysis, aPWV (with the use of the noninvasive Vicorder device; Skidmore Medical Ltd.), and a clinic BP measurement using a portable BP device (Omron) were performed followed by a blood collection. A finger-probe pulse oximeter (SpMet; Massimo Signal Extraction Technology) was used to measure methemoglobin concentrations in all participants during each study visit. Blood, urine, and saliva were collected for biochemical assessments. At the end of these measurements, participants consumed their first dose of juice. A subgroup of the first 34 enrolled patients also consented to a repeat of all vascular and BP measurements 3 h after juice ingestion. Participants consumed the juice once daily for the next 6 wk and returned for their final study visit at 6 ± 1 wk of study visit 1 and were expected to adhere to all of the same previsit rules.

### Primary endpoint

The primary outcome measure in this study was the absolute percentage change in the FMD response at 6 wk from baseline. FMD responses were measured at baseline and at 6 wk in both groups, and the absolute change in the response was calculated and compared between groups.

### Secondary endpoint measures

The principal secondary outcome measures included a within-group comparison of the FMD response at baseline and at 6 wk and assessment of the change at 6 wk from baseline of platelet reactivity. In addition, we explored other measures of vascular function including aPWV and pulse wave analysis and also assessed changes in nitric oxide metabolite concentrations.

### Exploratory endpoint measures

A number of additional prespecified exploratory measures were conducted including measurements of circulating inflammatory markers and markers of oxidative stress. These analyses were undertaken to gain some insight into potential mechanisms involved in any effects seen and were hypothesis generating only. In addition, a post hoc exploratory measure of oral-bacterial community profiling was conducted.

### FMD of the brachial artery

Ultrasonography was used to assess the FMD of the brachial artery and was performed by an experienced operator according to published protocols (20, 21) and as we have conducted previously (17).

### aPWV and aortic pulse wave analysis measurements

All arterial stiffness measurements were performed with the patient rested for 10 min in a supine position and awake. Six participants declined the measurement because of an inability to tolerate the carotid neck-cuff inflation. The pulse wave from the carotid and femoral arteries were simultaneously recorded with the use of an oscillometric method within the Vicorder device (22) and as described previously (23). The Vicorder software (Skidmore Medical Ltd.) was used to conduct an aortic transfer function to calculate the waveform and values for central BP as previously described (24).

### Urine and saliva sampling

Midstream urine samples were collected into sterile pots and aliquots were stored at −80°C pending the analysis of nitrite and nitrate concentrations with the use of ozone chemiluminescence. Unstimulated saliva was collected and centrifuged (14000 × *g*, 4°C, 10 min), and the supernatant fluid was stored at −80°C pending an analysis by ozone chemiluminescence.

### Oral microbiome profiling

Oral microbiome community profiling was conducted on samples collected at baseline and after 6 wk of treatment from 16 patients in the nitrate group and 14 patients in the placebo group (i.e., 32 and 28 samples, which made a total of 60 samples). DNA was extracted from the pellets for each saliva sample with the use of the GenElute Bacterial DNA Extraction Kit (Sigma-Aldrich). Extractions were conducted according to the manufacturer’s instructions with an additional lysis step to increase the recovery of Gram-positive bacterial DNA. Samples were incubated with a 45-mg/mL lysozyme solution at 37°C for 30 min. Samples were subjected to 16S ribosomal RNA gene polymerase chain reaction (PCR) and 454 pyrosequencing as previously described (25). An ∼500-bp region of the 16S ribosomal RNA gene (covering V1–V3) was PCR amplified from extracted DNA samples with the use of composite fusion primers comprising universal 16S primers (27FYM and 519R) along with GS-FLX Titanium Series adapter sequences (A & B, Roche) for 454 pyrosequencing with the use of the Lib-L emPCR (Roche) method. Previously described unique 12 base error-correcting Golay barcode sequences were incorporated into the forward primers (5′-CCATCTCATCCCTGCGTGTCTCCGACTCAG-NNNNN NNNNNNN-AGAGTTTGATYMTGGCTCAG-3′) to enable the pooling of samples in the same sequencing run. The appropriate barcoded A-27FYM and the B-519R (5′-CCTATCCCCTGTGTGCCTTGGCAGTCTCAG-GWATT ACCGCGGCKGCTG-3′) primers were used in PCRs with Extensor Hi-Fidelity PCR Mastermix (Thermo Scientific) (25).

### Blood sampling

Blood was collected into evacuated tubes (BD Bioscience) that contained EDTA (for chemiluminesence), 3.2% buffered sodium citrate (for aggregation assay and platelet flow cytometry), or a clot activator and gel (for serum cholesterol) through a 21-gauge butterfly needle inserted into an antecubital vein. For the measurement of nitrite and nitrate concentrations, blood samples were centrifuged immediately (1300 × *g*, 4°C, 10 min), and the supernatant fluid was collected and stored at −80°C pending an analysis with the use of ozone chemiluminescence. Serum cholesterol samples were sent to the St. Bartholomew’s Hospital biochemistry laboratory for analysis, and uric acid was measured with the use of a colorimetric kit according to the manufacturer’s instructions (Sigma-Aldrich). For plasma cyclic guanosine-5'-monophosphate (cGMP) determination, blood was collected into tubes containing the phosphodiesterase inhibitor, 3-isobutyl-1-methylxanthine (100 μmol/L; Sigma-Aldrich), and plasma cGMP concentration was measured with the use of an enzyme immunoassay kit in accordance with the manufacturer’s instructions (GE Healthcare). A number of other markers of oxidative stress and inflammation, i.e., plasma [CXCL1], high-sensitivity C-reactive protein (hs-CRP), and oxidized LDL, were measured with the use of specialized kits according to the manufacturers’ instructions.

### Measurement of nitric oxide–related species

Briefly, ozone chemiluminescence was used to determine total nitrate and nitrite (NO_x_) concentrations, and samples were added to 0.1 mmol/L vanadium (III) chloride in 1 mmol/L hydrochloric acid refluxing at 95°C under nitrogen. Nitrite concentrations were determined by the addition of samples to 0.09 mmol/L potassium iodide in glacial acetic acid under nitrogen at room temperature. [Nitrate] was calculated by the subtraction of [nitrite] from [NO_x_] as previously described (26).

Apparent total *N*-nitroso compounds (NOCs) were measured in urine with the use of a modification of methods that were previously validated (27, 28) with an Ecomedics CLD 88 Exhalyzer (Ecomedics). Urine samples were separated into three 500-μL tubes. A total of 100 μL urine was mixed with 500 μL of a 5% (weight:volume) sulfanilamide solution to remove nitrite and injected into a purge vessel that was kept at 60°C and filled with a standard tri-iodide reagent (38 mg I_2_ was added to a solution of 108 mg KI in 1 mL H_2_O; to this mixture, 13.5 mL glacial acetic acid was added) to determine total NOCs. To determine ferricyanide stable compounds (nitrosyl iron) to a sample of 100 μL a 10 mmol/L aqueous solution of K_3_Fe(CN)_6_ was added before analysis. Total nitrosyl iron compounds were determined as the difference between apparent total nitroso compounds with and without the addition of K_3_Fe(CN)_6_.

### Platelet flow cytometry

Whole blood flow cytometry was used to measure platelet P-selectin with the use of a modification of previously published protocols and recommendations (16, 29, 30). The platelet population was identified via labeling with a CD42b (1:25; eBioscience) monoclonal antibody conjugated to allophycocyanin and a CD62P (P-selectin; eBioscience) monoclonal antibody conjugated to fluorescein isothiocyanate, which was used to determine the P-selectin expression. Isotype controls for CD62P and CD42b were used to ensure antibody specificity. All samples were run in duplicate. Samples were incubated at room temperature for 20 min with phosphate-buffered saline, ADP (10 μmol/L; Labmedics), collagen (3 μg/mL; Takeda), or adrenaline (10 μmol/L; Labmedics) before fixing with 1% paraformaldehyde (Sigma-Aldrich) and analyzed with the use of a Becton Dickinson FACSCalibur flow cytometer (Becton Dickinson). A total of 10,000 platelets were acquired in the CD42b-positive region. Results were expressed as the percentage of platelets positive for P-selectin (16).

### Platelet-monocyte aggregate expression

Platelet-monocyte aggregates (PMAs) were determined with the use of a modification of previously published protocols (30) with antibodies that were selective for platelet CD42b, and monocyte-marker CD14 (BD Bioscience) samples were analyzed with the use of a Becton Dickinson Fortessa flow cytometer (Becton Dickinson). Isotype controls were conducted.

### Measurements of plasma oxidized LDL concentration, uric acid, CXCL1, and hs-CRP concentration

A sandwich ELISA (Mercodia) that was based on the monoclonal antibody mAB-4E6 as described previously (31) was used to measure plasma oxidized LDL concentrations. CXCL1 concentrations in plasma samples were determined with the use of a human CXCL1/GROα DuoSet ELISA Development kit according to the manufacturer’s guidelines (R&D Systems). Plasma hs-CRP concentrations were measured with the use of a CRP ELISA kit in accordance with the manufacture’s guidelines (eBioscience). The concentration of hs-CRP is a predictor of a cardiovascular event, and patients can be stratified into low-risk (<1.0 mg/L), moderate-risk (1.0–3.0 mg/L), and high-risk (>3.0 mg/L) groups (32). Data obtained from the measurement of hs-CRP was classified accordingly for analysis. Uric acid concentrations were determined with the use of a commercially available kit (Sigma-Aldrich) and per the manufacturer’s guidelines.

### Statistical analysis

The study was powered for the primary outcome measure of the percentage change in the FMD response at 6 wk from baseline. With the assumption of an average ± SD improvement of FMD of 1.1 ± 1.45% and the assumption of no change in the placebo group, 30 volunteers were required within each group for a statistical power of 0.8 at a significance level of α = 0.05. Thus, a total of 60 patients were required. We assumed a potential 10% dropout rate (as per our in-house experience), which resulted in a total recruitment target of 66 patients. These calculations were based on a number of previous published observations. Improvements in FMD were noted after 6 wk of artichoke juice intake (33) and also by 2 additional studies with walnuts (34) and one recent meta-analysis (35) that assessed the effects of chronic polyphenol dietary interventions. The averaged improvement across all of these studies was an absolute increase of 1.1% (with an average absolute FMD response of 5%), which approximated to an improvement of 25%. These proposed numbers also provided sufficient power for the outcome measure of improved FMD for within-group comparisons. With the use of the conservative estimates of an expected increase in FMD of 25% from 5% to 6.7% after 6 wk of beetroot juice intake in this population, a sample size of 20 was needed to detect a difference with a power of 0.8 at significance level of α = 0.05 [assuming an SD of ultrasound FMD of 2.5 (21)] for a within-group comparison with baseline.

Baseline demographic and clinical variables were summarized for each group of the study. Statistical comparisons for the primary outcome measure were between the dietary nitrate-treated and placebo-treated group. An unpaired *t* test was used for the comparison of the change in the FMD response between groups. For within-group comparison, a paired *t* test was used. All *P* values were 2 tailed.

For the platelet measures, ANOVA with Bonferroni posttests were applied to compare the baseline with the 6 wk time point for the within-group comparison. For data with skewed distribution data (platelet P-selectin expression), the Kruskal-Wallis test was used with Dunn’s post-tests for within-group comparisons. All data were analyzed with the use of Prism software version 5 (Graphpad). All exploratory data are expressed as per Consolidated Standards of Reporting Trials guidelines with data showing a normal distribution as means ± SDs, data with a skewed distribution as medians and IQRs, and effect sizes (i.e., differences from baseline) shown as means with 95% CIs.

For the bacterial community analysis, the data were analyzed with the use of the mothur pipeline. Sequences were filtered to exclude sequences that were shorter than 440 bases. Operational taxonomic units (OTUs) were built at a distance cutoff of 0.15 and identified with the use of the mothur Bayesian classifier with reference to the Human Oral Microbiome Database reference dataset (36). The thetaYC metric (37) was used to generate distance matrices from the sequence libraries, which were visualized as principal coordinates analysis plots. Three-dimensional principal coordinates analysis plots were generated in the R program (r-project.org) with the use of the rgl package. Treatment groups were compared by means of an analysis of molecular variance (38), which currently stands as the most-appropriate methodology of choice for the assessment of change in a microbial community analysis (39), and OTUs that were differentially associated with treatment groups were determined by means of the Metastats program (40) and visualized by means of LefSe (41).

## RESULTS

In this study, 67 participants completed both study visits. Two participants withdrew consent after screening at the time of visit 1. One participant wished to unblind the intervention before continuing, and the other participant felt unwell at the time of the first visit. The primary outcome measure of FMD was conducted in all participants; however, full data sets for the analysis were generated for only 65 participants (32 volunteers who received the placebo and 33 volunteers who received dietary nitrate), which was attributed to a loss of brachial artery measurements as a consequence of inadequate electrocardiographic gating for one participant and because of a file corruption for another participant (Figure 1).

The nitrate content of the active treatment juice was 24.2 ± 7.7 mmol/L, which gave ∼6.0 mmol nitrate in a 250-mL daily dose, with the placebo juice at 0.053 ± 0.12 mmol/L, which gave ∼0.001 mmol nitrate/d. Nitrite was below the limits of detection in both interventions (i.e., <50 nmol/L). The interventions were well tolerated and without adverse effects apart from beeturia that was noted in both groups.

There were no differences in baseline demographics between the 2 groups of the whole cohort (**Table 1**) or the subgroup (**Supplemental Table 1**). Methemoglobin concentrations were unaltered by either treatment (**Supplemental Table 2**). Total apparent NOCs were similar at baseline with an ∼40-fold increase in the nitrate group. The treatment of samples to remove all iron-nitrosyl resulted in a complete loss of signal, which suggested a predominance of iron-nitrosyl (Supplemental Table 1). Baseline lipid concentrations were similar between the groups (Table 1, Supplemental Table 1) and unaltered by either intervention (Supplemental Table 2).

**TABLE 1:** Baseline characteristics^1^

	Group	
Variable	Nitrate (*n *= 33)	Placebo (*n *= 34)
Age, y	53.3 ± 10.1^2^	53.2 ± 11.8
Sex, M:F, *n*	12:21	12:22
BMI, kg/m^2^	26.8 ± 4.9	26.7 ± 5.1
Baseline SBP, mm Hg	125.2 ± 15.1	122.7 ± 15.2
Baseline DBP, mm Hg	76.3 ± 8.6	78.1 ± 11.2
Total cholesterol, mmol/L	6.7 (6.0–7.3)^3^	6.7 (6.3–7.5)
LDL, mmol/L	4.3 (3.7–5.1)	4.4 (3.9–5.1)
Treated hypertensives, *n*	9	5
Medications, *n*		
ACE inhibitor/ARB	3	4
Calcium antagonists	4	4
β-Blocker	1	0
Thiazide diuretic	5	1

1 ACE, angiotensin-converting enzyme; ARB, angiotensin receptor blocker; DBP, diastolic blood pressure; SBP, systolic blood pressure.

2 Mean ± SD (all such values for normally distributed variables).

3 Median; IQR in parentheses (all such values for non–normally distributed variables).

### Enterosalivary circuit is intact in hypercholesterolemia

**FIGURE 2:**
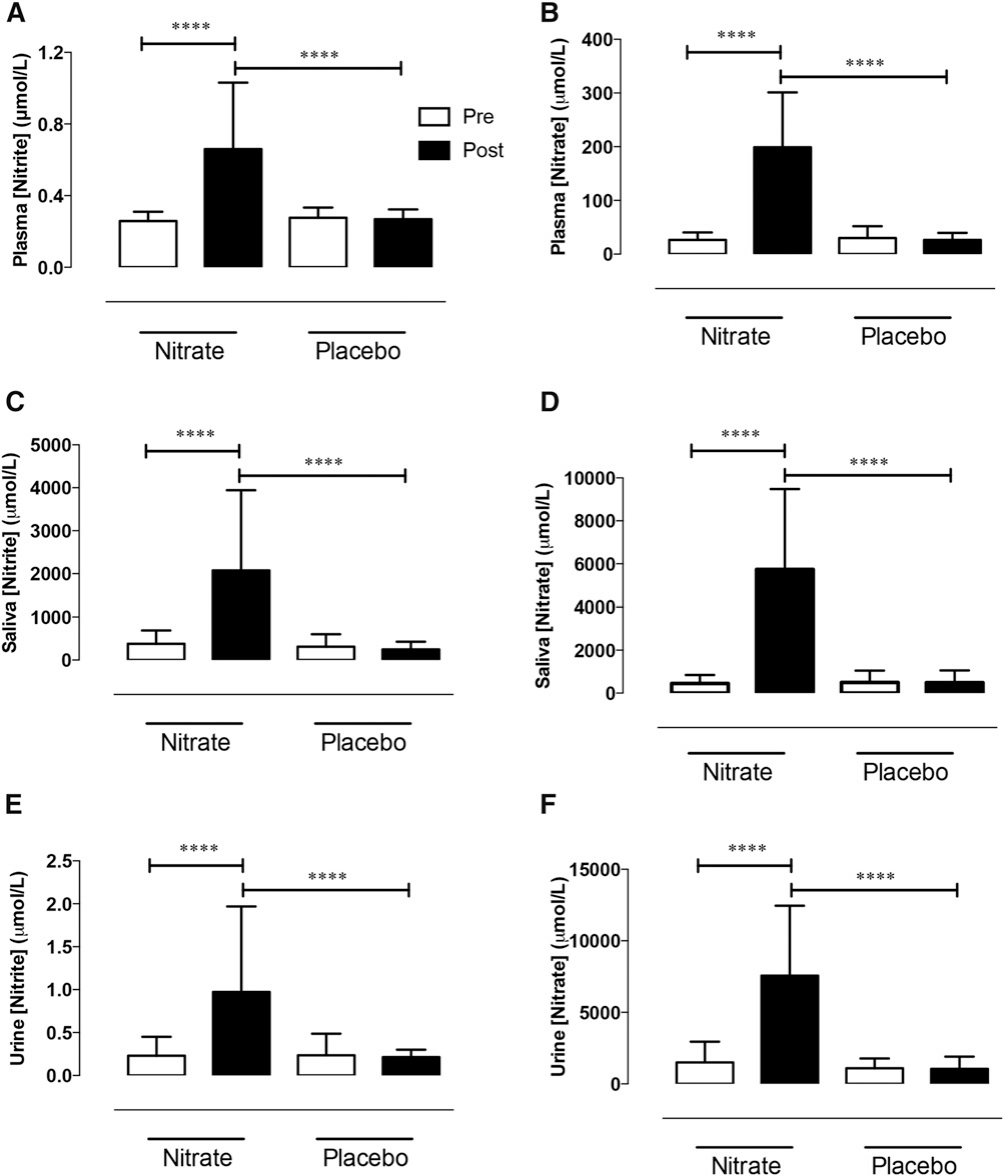
Dietary nitrate elevates plasma, salivary, and urinary nitrite and nitrate concentrations in hypercholesterolemic patients. Mean ± SD effects of 6 wk of dietary nitrate consumption (250 mL nitrate-rich juice/d) or placebo consumption (250 mL nitrate-depleted juice/d) on nitrite and nitrate concentrations in plasma (A and B), saliva (C and D) and urine (E and F), respectively. *n* = 33 in the nitrate group; *n* = 32 in the placebo group. ****Significant, *P* < 0.001 (1-factor ANOVA with Bonferroni posttests). There were no significant differences in any comparisons between baseline measures in the nitrate and placebo groups.

There were no differences in baseline plasma cGMP concentrations [nitrate group (15.47 ± 2.91 nmol/L) compared with placebo group (15.28 ± 3.92 nmol/L), *P* = 0.82] or NOx concentrations between groups (**Figure 2**). After both 3 h and 6 wk of dietary nitrate plasma, salivary and urinary [nitrate] and [nitrite] were elevated (Figure 2, **Supplemental Table 3**) with no changes in other serum electrolytes (baseline and 6-wk data; Supplemental Table 1).

### Inorganic nitrate improves vascular function

There was no difference in the baseline brachial artery diameter or time taken for the peak FMD response between groups at either the 6-wk (**Table 2**) or 3-h time point (**Table 3**). The CV for the baseline diameter measurement was 2.4 ± 1.6% .

**TABLE 2:** Effects of dietary nitrate after 6 wk of once-daily treatment on BP and vascular function^1^

	Nitrate (*n* = 33)				Placebo (*n* = 32)				
	Baseline	6 wk	Difference	*P*	Baseline	6 wk	Difference	*P*	*P*-between group
Ultrasound									
Baseline brachial artery diameter, mm	3.8 ± 0.6^2^	3.8 ± 0.6	0.02 (−0.03, 0.08)^3^	0.19	3.7 ± 0.7	3.7 ± 0.6	−0.02 (−0.7, 0.04)	0.57	0.33
ΔAbsolute diameter in response to flow, mm	0.17 ± 0.07	0.21 ± 0.08	0.03 (0.01, 0.05)	0.004	0.17 ± 0.07	0.15 ± 0.07	−0.01 (−0.02, −0.00002)	0.05	0.0003
Time to peak diameter, min	6.9 ± 0.3	7.0 ± 0.2	0.04 (−0.06, −0.1)	0.18	6.9 ± 0.3	6.9 ± 0.3	−0.01 (−0.2, 0.1)	0.87	0.52
PWA									
Augmentation index, %	28.7 ± 7.3	26.4 ± 7.8	−2.4 (−4.5, −0.2)	0.04	25.8 ± 7.4	27.4 ± 8.3	1.5 (−0.7, 3.8)	0.17	0.013
PWV, m/s	8.3 ± 1.4	8.0 ± 1.1	−0.2 (−0.4, −0.03)	0.02	8.0 ± 1.1	8.1 ± 1.1	0.04 (−0.2, 0.3)	0.68	0.063
Clinic blood pressure									
SBP, mm Hg	125.2 ± 15.1	121.1 ± 12.2	−4.1 (−6.8, −1.4)	0.004	122.7 ± 15.2	120.1 ± 15.4	−2.7 (−6.9, 1.5)	0.22	0.57
DBP, mm Hg	76.3 ± 8.6	75.0 ± 8.3	−1.5 (−3.4, −0.3)	0.19	78.1 ± 11.2	76.2 ± 12.8	−1.6 (−3.8, 0.6)	0.12	0.97
Heart rate, beats/min	67 ± 8	65 ± 9	−1.5 (−4.0, 1.1)	0.18	66 ± 8	65 ± 8	−1.2 (−3.2, 0.8)	0.3	0.88

1 Data are shown for ultrasound measures including the resting brachial artery diameter, absolute change in diameter, time to peak diameter, and percentage of flow-mediated dilatation. The PWA is depicted as an augmentation index, and aortic PWV, blood pressure, and heart-rate data are shown. Values are averages at baseline and 6 wk. *P* values shown are for paired *t* tests before and after 6 wk of treatment or placebo intake, and an unpaired *t* test was used for the comparison of differences between groups. BP, blood pressure; DBP, diastolic blood pressure; PWA, pulse wave analysis; PWV, pulse wave velocity; SBP, systolic blood pressure.

2 Mean ± SD (all such values for differences between baseline and 6 wk for each group).

3 Mean; 95% CI in parentheses (all such values for differences between baseline and 6 wk for each group).

**TABLE 3:** Acute effects of dietary nitrate 3 h after first dose on BP and vascular function^1^

	Nitrate (*n* = 17)				Placebo (*n* = 17)				
	Baseline	3 h	Difference	*P*	Baseline	3 h	Difference	*P*	*P*-between group
Ultrasound									
FMD, %	5.3 ± 2.3^2^	6.8 ± 2.3	1.5 (0.4, 2.7)^3^	0.01	4.8 ± 2.0	4.9 ± 2.1	0.1 (−0.9, 1.1)	0.86	0.05
Baseline brachial artery diameter, mm	3.7 ± 0.5	3.7 ± 0.5	−0.02 (−0.1, 0.05)	0.86	3.9 ± 0.7	3.9 ± 0.7	−0.02 (−0.1, 0.1)	0.44	0.89
ΔAbsolute diameter in response to flow, mm	0.19 ± 0.07	0.25 ± 0.07	0.2 (−0.2, 0.6)	0.50	0.18 ± 0.07	0.18 ± 0.06	−0.0004 (−0.04, 0.03)	0.79	0.24
Time to peak diameter, min	6.9 ± 0.3	6.9 ± 0.3	0.05 (−0.2, 0.3)	0.63	6.9 ± 0.3	6.8 ± 0.3	−0.08 (−0.3, 0.1)	0.35	0.33
PWA									
Augmentation index, %	30.1 ± 8.0	27.6 ± 7.5	−2.5 (−4.4, −0.6)	0.015	25.4 ± 9.1	2.0.4 ± 9.4	3.0 (−0.5, 0.02)	0.001	<0.0001
PWV, m/s	8.4 ± 1.8	8.1 ± 1.6	−0.3 (−0.5–0.03)	0.023	8.5 ± 1.0	8.8 ± 1.4	0.2 (−0.4, 0.9)	0.73	0.15
Clinic blood pressure									
SBP, mm Hg	129.4 ± 17.8	122.0.2 ± 18.2	−7.2 (−12.9, −1.4)	0.02	127.1 ± 15.7	125.3 ± 12.7	−3.1 (−8.3, 2.1)	0.43	0.27
DBP, mm Hg	78.6 ± 9.4	75.5 ± 8.8	−2.6 (−5.4, 0.2)	0.03	82.6 ± 11.8	79.3 ± 12.9	−3.4 (−5.8, −0.9)	0.01	0.68
Heart rate, beats/min	68 ± 10	70 ± 11	3.0 (−0.1, 6.2)	0.26	68 ± 7	68 ± 8	−0.2 (−2.2, 1.9)	0.88	0.59

1 Data shown are for ultrasound measures including the resting brachial artery diameter, absolute change in diameter, time to peak diameter, and percentage of FMD. The PWA is depicted as an augmentation index, and aortic PWV, blood pressure, and heart-rate data are shown. Values are averages at baseline and 6 wk. *P* values shown are for paired *t* tests before and after 3 h of treatment or placebo intake, and an unpaired *t* test was used for the comparison of differences between groups. BP, blood pressure; DBP, diastolic blood pressure; FMD, flow-mediated dilatation; PWA, pulse wave analysis; PWV, pulse wave velocity; SBP, systolic blood pressure.

2 Mean ± SD (all such values for differences between baseline and 3 h for each group).

3 Mean; 95% CI in parentheses (all such values for differences between baseline and 3 h for each group).

**FIGURE 3:**
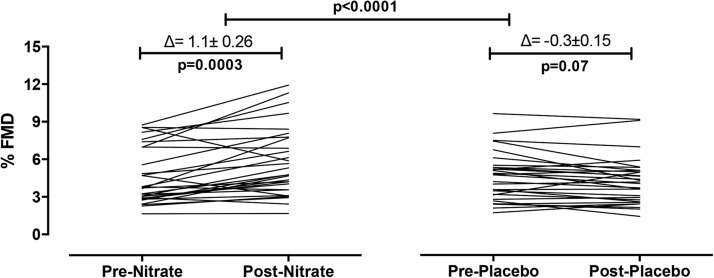
Dietary nitrate improves vascular function in hypercholesterolemic patients. Mean ± SD effects of 6 wk of dietary nitrate consumption (250 mL nitrate-rich juice/d) or placebo consumption (250 mL nitrate-depleted juice/d) on FMD. *n* = 33 in the nitrate group; *n* = 32 in the placebo group. Baseline and 6-wk data are shown for groups before and after intake of nitrate-rich juice and nitrate-depleted placebo juice. *P* values shown are for within-group comparisons with the use of paired *t* test for the comparison of the baseline FMD with the response after 6 wk of intervention. Comparison between groups are shown with *P* values for the change from baseline with the use of an unpaired *t* test. FMD, flow-mediated dilatation.

There was a significant improvement in the primary outcome measure of the change in FMD at 6 wk of intervention with a ∼24% increase in the nitrate-treated group and a trend for a small decrease from baseline (∼6%) in the placebo group (*P* = 0.07) with the %change in FMD in the nitrate group significantly different from the percentage of change in the placebo group (*P* < 0.0001) (Table 2, **Figure 3**). These results were accompanied by an improved augmentation index compared with at baseline and in the placebo group (Table 2) and an improved aPWV compared with in the placebo group (*P* = 0.013) with a trend for significance comparison with the placebo group (*P* = 0.063). A similar profile of effects on all vascular variables was also evident at 3 h after the first dose of dietary nitrate (Table 3). The CV for the %FMD response was 14.3 ± 10.3%.

**FIGURE 4:**
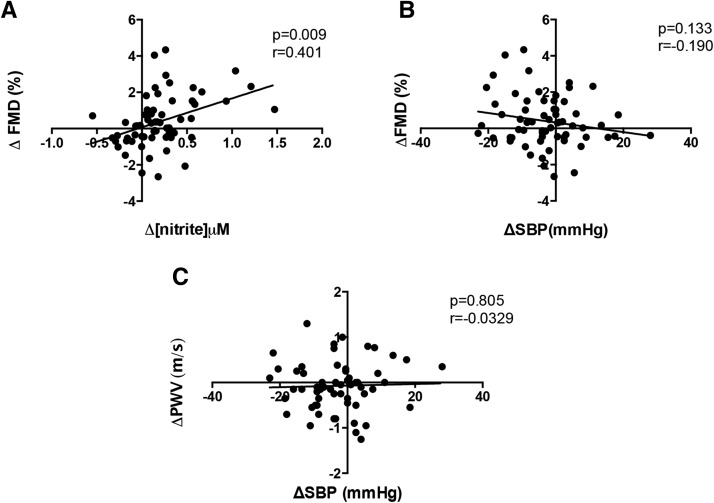
Associations between plasma nitrite concentrations and vascular function measures. Changes were determined from baseline to the 6-wk time point in FMD relative to changes in plasma nitrite concentration (A) and changes in blood pressure (SBP) (B). (C) Changes in vascular stiffness aortic PWV are plotted against changes in SBP. Associations were determined with the use of a Pearson’s correlation coefficient assessment. The data show values for *n* = 33 in the nitrate group and *n* = 32 in the placebo group for each correlation analysis. FMD, flow-mediated dilatation; SBP, systolic blood pressure. PWV, pulse wave velocity.

Although small decreases in systolic BP and diastolic BP but not heart rate (Tables 2 and 3) were evident, differences from baseline were not significantly different between groups, and post hoc analyses indicated no correlation between changes in vascular function and systolic BP but a direct correlation with the change in plasma [nitrite] (**Figure 4**).

### Inorganic nitrate treatment reduces PMA levels and P-selectin expression

**FIGURE 5:**
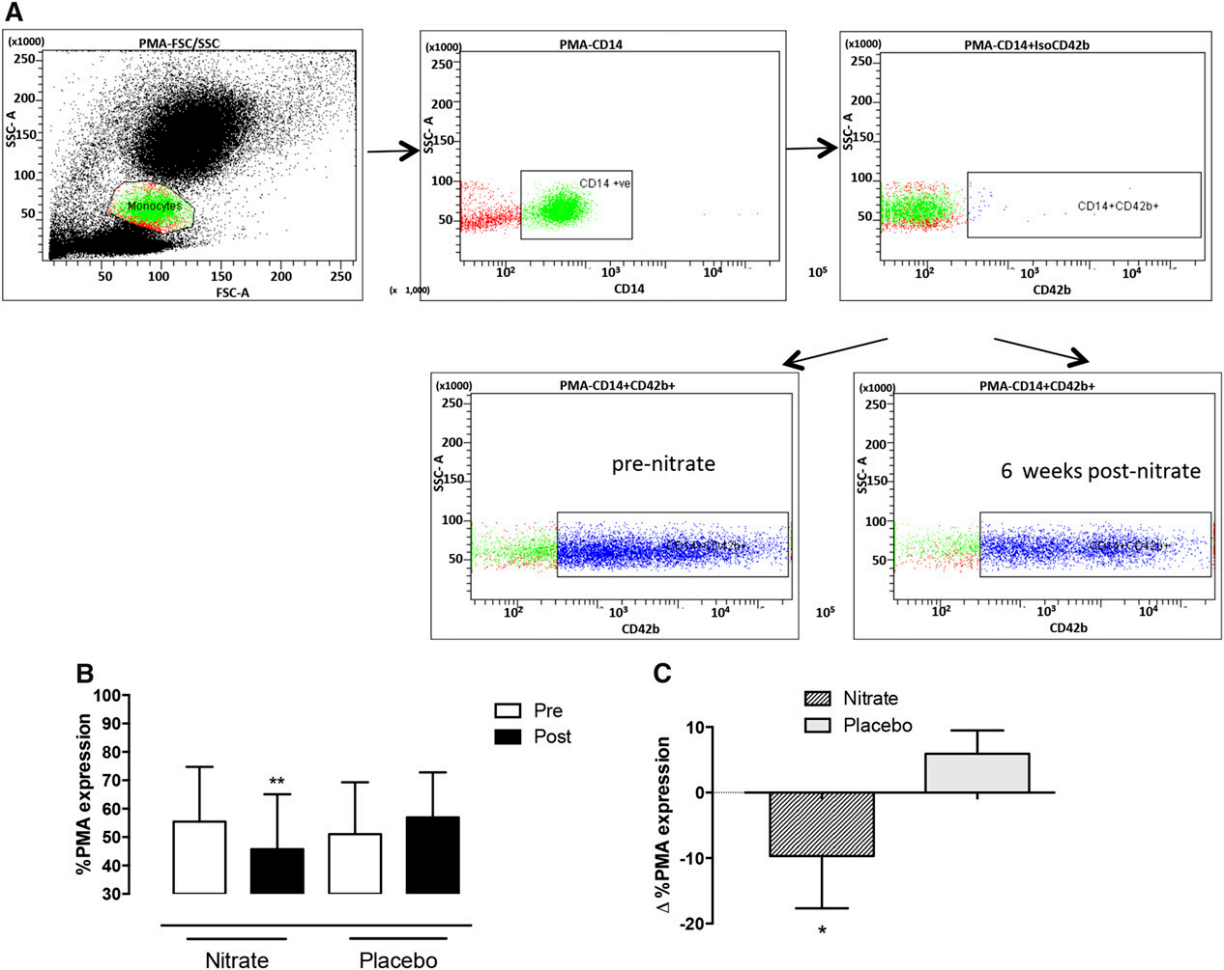
Dietary nitrate decreases platelet monocyte aggregate concentrations. Mean ± SD effects of 6 wk of dietary nitrate consumption (250 mL nitrate-rich juice/d) or placebo consumption (250 mL nitrate-depleted juice/d) on flow-cytometry measures of PMA (A) and the percentage of PMA formation for groups before and after intake of nitrate-rich juice and placebo juice (B). (C) Change in % PMA formation over 6 wk in the 2 groups expressed as mean (95% CI). Data shown are *n* = 25 for the nitrate group and *n* = 27 for the placebo group. **Significant for within-group comparisons of baseline compared with postnitrate consumption, *P* < 0.01 (paired Student’s *t* test). *Significant for the comparison between groups, *P* < 0.05 (unpaired *t* test). PMA, platelet-monocyte aggregate.

After 6 wk of dietary nitrate intake, PMA levels were reduced compared with after placebo intake (*P* = 0.02) (**Figure 5**) with a trend for a reduction in platelet P-selectin concentrations (**Supplemental Figure 1**) and reduced P-selectin expression in response to ADP and adrenaline but not to collagen (Supplemental Figure 1).

### Bacterial community profiling

**FIGURE 6:**
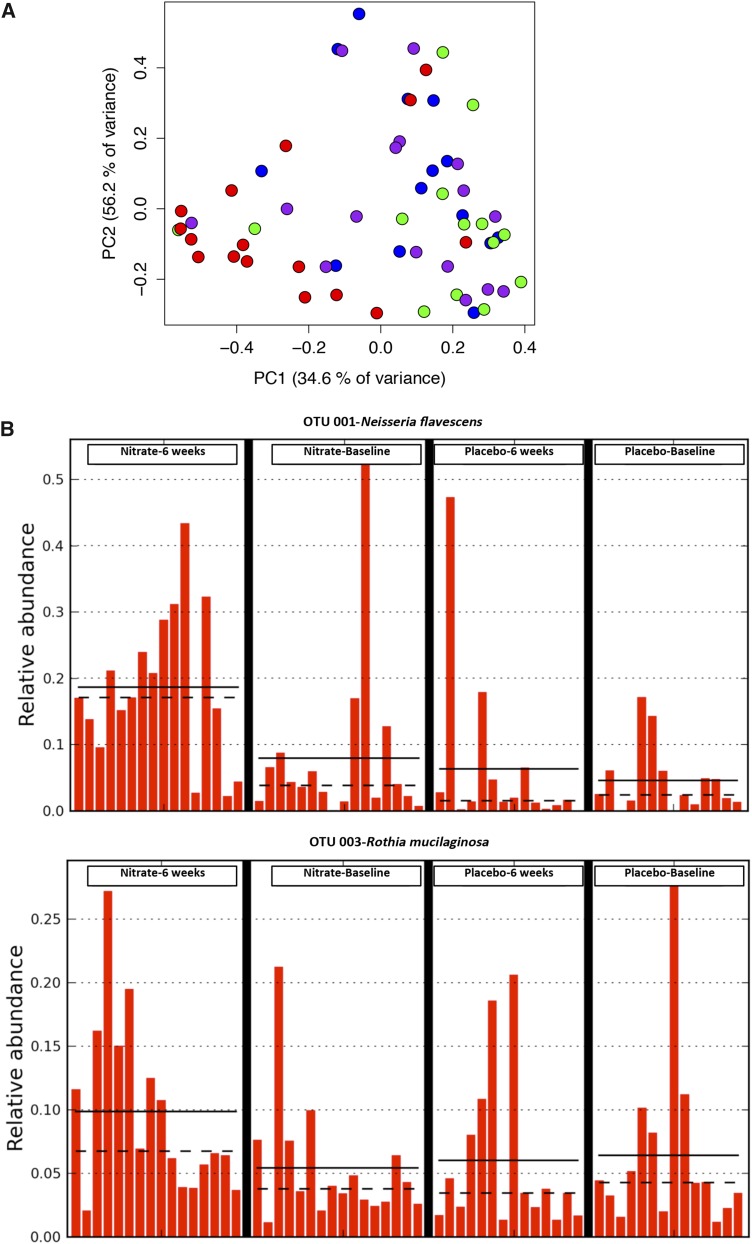
Dietary nitrate results in changes in the oral microbiome. The use of a bacterial community profile analysis by means of the mothur pipeline (16S ribosomal RNA gene) identified 78 different OTUs, the proportions of which were altered by dietary nitrate treatment. The statistical analysis was conducted with the use of an AMOVA (38) for assessment of the change between groups in the oral microbial community (39). The AMOVA gave *P* < 0.001 for the within-group comparison between baseline and post-nitrate and *P* = 0.001 for the between-group comparison of post-placebo compared with post-nitrate. Of these OTUs, those that had their numbers increase post-treatment and that made up >1% of the post-treatment community were *Neisseria flavescens* and *Rothia mucilaginosa*. (A) Plot depicts a principal coordinate analysis that was based on the ThetaYC metric, which compared the structure of the communities (PC1 = 34.6% of variance explained; PC2 = 56.2%). Colored circles represent the 2 groups of the study. Blue and green circles represent baseline and after 6 wk of placebo intake, respectively, and purple and red circles represent baseline and after 6 wk of once-daily intake (5 mmol) of dietary nitrate juice, respectively. (B) Representation of the relative abundances of *R. mucilaginosa* and *N. flavescens* at baseline and postnitrate or postplacebo treatment of 6 wk. Data are shown for baseline and 6-wk values for *n* = 16 in the nitrate group and *n* = 14 in the placebo group. Solid lines denote group means, and dotted lines denote group medians. AMOVA, analysis of molecular variance; OTU, operational taxonomic unit.

Changes in [NOx] at 6 wk were associated with alterations in the oral microbial community (**Figure 6**) with a significant shift in the structure of the bacterial community after dietary nitrate intake that was not evident in the placebo group. The proportions of 78 OTUs were significantly altered in the dietary nitrate group, and the identity of the OTUs that made up >1% of the community and the proportions of which increased after treatment were *Neisseria flavescens* and *Rothia mucilaginosa* (Figure 6). The proportions of *N. flavescens* were elevated significantly after nitrate intake compared with after placebo intake (*P* < 0.01); however, the differences did not reach significance for *R. mucilaginosa*.

### Measurements of plasma uric acid, oxidative stress, and inflammatory markers

Plasma [uric acid] and the inflammatory marker [CXCL1] displayed a trend for attenuation after dietary nitrate (Supplemental Table 3). Oxidized LDL concentrations were similar at baseline with a trend for a greater reduction at 6 wk after dietary nitrate intake, although reductions were evident in both groups (**Supplemental Table 4**). Overall, no difference in hs-CRP concentrations was evident (Supplemental Table 3); however, stratification at baseline according to low, intermediate, and high hs-CRP indicated a trend for suppression with nitrate in subjects with intermediate to high concentrations (Supplemental Table 4). No significant differences were shown for comparisons between groups of the change from baseline of each of the markers. The assay variability for CXCL1, oxidized LDL, hs-CRP, and uric acid was 1.7 ± 1.5%, 3.8 ± 3.2%, 4.8 ± 4.9%, and 3.8 ± 4.1%, respectively.

## DISCUSSION

In this 6-wk trial of daily inorganic nitrate (beetroot juice) ingestion compared with intake of a low-nitrate placebo in patients with hypercholesterolemia, a rise in circulating nitrite was associated with a ∼24% improvement in the primary outcome measure of the FMD response together with improvements in measures of arterial stiffness. These improvements compared with a ∼6% decline in the FMD response in the placebo cohort. This effect was also associated with reductions in circulating PMA numbers and reduced platelet P-selectin expression. Together, these data intimate that dietary nitrate might be useful in improving vascular and platelet functions in hypercholesterolemic patients.

Dietary nitrate treatment elevated circulating concentrations of both nitrate (∼7.5-fold) and nitrite (∼2.5-fold), indicating an intact enterosalivary circuit in hypercholesterolemic patients. Once absorbed, inorganic nitrate consumed from the diet is extracted from the blood by the salivary glands (42) and consequently secreted into the oral cavity (43). The commensal bacteria reduce the nitrate to nitrite. This nitrite is swallowed and appears within the circulation (21, 44). We have shown that this circuit remained functional over the 6-wk once daily regimen with no evidence of tachphylaxis. Dietary nitrate caused a 7.7-fold increase in circulating nitrate concentrations after 3 h with an identical rise after 6 wk. These changes were associated with rises in plasma [nitrite] of 2.0- and 2.5-fold at 3 h and 6 wk, respectively; the increase in plasma nitrite concentrations possibly suggesting a progressively increased nitrate reduction with persistent dietary nitrate ingestion.

This apparent improvement in circulating nitrite concentrations was associated with a shift in the bacterial community within the oral cavity with raised numbers of *N. flavescens* and *R. mucilaginosa*, which are species that exhibit denitrification activity (45, 46). These findings intimate a shift in the oral microbiome in favor of organisms that are capable of nitrate reduction. In healthy volunteers, the nitrite:nitrate in the oral cavity is 0.3 (47). In this study, the ratio of the change from baseline of nitrite:nitrate in the saliva 3 h after nitrate administration was 0.35 ± 0.07, and at the 6-wk time point, it was 0.41 ± 0.06; these values support the hypothesis of an increased nitrate reduction at 6 wk. *N. flavescens* and *R. mucilaginosa* are normally regarded as obligate aerobes, whereas the biofilms that form on oral surfaces rapidly become anaerobic with time, and plaque bacterial communities are dominated by facultative and obligate anaerobes. It is interesting that *Neisseria*, in particular, can grow under anaerobic conditions with nitrate or nitrite acting as an electron acceptor (45, 46). *Neisseria* was recently reported to be one of the principal oral bacterial genera responsible for the nitrate reduction in healthy volunteers (48). Additional prospective studies that assess whether persistent dietary nitrate ingestion might provide improved nitrate bioactivation in the longer term are warranted.

In this study, we showed that dietary nitrate ingestion resulted in an increase in FMD (∼24%) and, thus, an improvement of vascular function. The beneficial effects of nitrate were evident at 3 h after the first dose as well as at 6 wk, which indicated no tachyphylaxis over time and suggested that the mechanisms underlying these effects involve the acute modification of pathways that result in immediate functional effects. Our findings differ from recent observations in patients with diabetes where no effect of daily dietary nitrate (2 wk of 7.5 mmol/d) was evident (18) but are in accord with our own (albeit unpowered) observations in hypertensive patients who consumed a single dietary nitrate dose (∼6 mmol) for 4 wk (17) where dietary nitrate improved FMD responses. The reasons for the differences were unlikely to be related to the dose (although the study by Benjamin et al. (18) in patients with diabetes used a dose that was ∼1.5 mmol higher than that used in the current study) but may have been related to the duration of treatment, a resistance to inorganic nitrate in patients with diabetes, or insufficient power.

Dietary nitrate treatment also resulted in modest improvements in both the aPWV and augmentation index, both of which are measures of arterial stiffness. Previous assessments in aged mice showed that nitrite supplementation for 3 wk resulted in the destiffening of the aorta because of a reduced concentration of oxidative stress-induced advanced glycation end products (49). Whether this mechanism might underlie the effects seen in this study is uncertain but warrants assessment. It is possible that the improvements in vascular function, particularly in the aPWV but also the FMD, were secondary to decreases in BP. However, our post hoc correlation analyses showed no association between the change in vascular function and BP. Additional studies powered to apply multivariate analyses to interrogate these issues more closely are warranted.

We have previously speculated that the provision of nitrite as a substrate to certain nitrite reductases, particularly the enzyme xanthine oxidoreductase (XOR), not only results in NO generation but might also result in reductions of oxidative stress by competing for electrons that are required for oxygen reduction (50). Conventional XOR activity (i.e., the conversion of xanthine to uric acid) occurs at the molybdenum-binding site of the enzyme, which is also the site of nitrite reduction (51, 52). Our exploratory analyses herein showing apparent reduced concentrations of uric acid with nitrate treatment supports the suggestion of an effect at the level of the XOR enzyme. High concentrations of xanthine prevent nitrite reduction by XOR (53); however, whether nitrite might compete with xanthine at the molybdenum site of the enzyme is unknown and worth investigating.

We showed no evidence of alterations in lipid concentrations in contrast with preclinical studies in hypercholesterolemic C57BL/6J mice (54) and a small study in hypercholesterolemic patients (55). The reasons for this difference are uncertain, but our study was not powered to detect small reductions in LDL or triglyceride, and larger studies to assess this difference may be worthwhile.

Dietary nitrate treatment was associated with small but significant reductions in markers of platelet activation. Such an effect may be beneficial because activated platelets are thought to play a role in the progression of atherosclerotic disease in humans (56, 57). PMA assessment has been proposed to be a superior marker of platelet activation compared with surface P-selectin, and preclinical studies in mice have suggested that targeting PMA may yield therapeutic benefit because these aggregates play a crucial role in inducing endothelial dysfunction and consequent atheroma formation (58). Although the concentrations of P-selectin in unstimulated platelets were not significantly altered by dietary nitrate treatment, a small but significant reduction in P-selectin expression in response to ex vivo treatment with platelet activating stimuli suggest that reduction of stimulated P-selectin expression may underlie the reduced numbers of PMA (these aggregates form because of an interaction between platelet P-selectin and monocyte P-selectin glycoprotein ligand-1). NO lowers platelet reactivity through reductions in P-selectin expression (59); this effect has been implicated in nitrate- and nitrite-induced repression of platelet reactivity in humans (16, 60). A prospective study designed to test this directly is warranted.

The exact molecular mechanisms that underlie the improvements in vascular function are uncertain. However, it is thought that the reduced bioavailability of NO in hypercholesterolemia relates to an increased oxidative stress (61, 62). This relation, in turn, is thought to result in the scavenging of NO that triggers systemic inflammation. Because the FMD response is thought to be due in part to endothelium-derived NO release (63, 64), the increase in response might reflect an improvement in NO activity that is due to reductions in inflammation-induced oxidative stress and NO scavenging. Our analyses indicated that, although hs-CRP was not different in the whole cohort, in individuals with high baseline concentrations, a trend for a reduction was evident. We also observed a trend for a reduction of neutrophil chemokine CXCL1, which is a chemokine that has been implicated in human atherosclerotic disease (65), although concentrations of oxidized LDL, as a marker of oxidative stress, were not different between the groups. Additional prospective and appropriately powered studies that are designed to test whether dietary nitrate reduces inflammation and oxidative stress in hypercholesterolemia are required.

The safety profiles of dietary nitrate and nitrite have been much debated particularly with respect to methemoglobin concentrations and associations of dietary nitrite consumption and endogenous *N*-nitrosamine formation with gastric cancer (66). In this study, there was no evidence of methemoglobinemia and a small increase in apparent total NOC after nitrate. However, the concentrations of the latter fell within the range observed in healthy individuals and were lower than those thought to be associated with high risk of intestinal cancer. A major limitation of the NOC measurements was that we could not exclude the possibility that the iron-nitrosyl species in the urine were formed after sample collection. The pH of urine is generally slightly acidic and will favor NO release from nitrite and, thus, the potential for nitrosation of any free haem groups within the urine. Additional studies are warranted to clarify these issues.

We did not measure the effect of dietary nitrate on direct NO-stimulated increases in blood flow, which have conventionally been assessed with the use of glyceryltrinitrate. Therefore, we could not exclude the possibility that changes in the reactivity of the underlying smooth muscle might also have contributed to the improved vascular function. In addition, a number of mechanistic and exploratory analyses were conducted for which the study was not powered, including all of the inflammatory mediator, apparent NOC, and microbiome analyses. Additional prospective studies that are powered for each of these outcome measures are required to confirm the hypotheses proposed.

In conclusion, this study supports the use of dietary nitrate as a safe, well-tolerated, and potentially powerful prevention strategy in CVD in individuals with early vascular dysfunction. This strategy is evidenced by improvements in FMD, vascular stiffness, and the platelet inflammatory profile. Long-term outcome studies are required to test the merits of a dietary nitrate strategy.

## Footnotes

9 Abbreviations used: aPWV, aortic pulse wave velocity; BP, blood pressure; cGMP, cyclic guanosine-5′- monophosphate; CVD, cardiovascular disease; FMD, flow-mediated dilatation; hs-CRP; high-sensitivity C-reactive protein; NOC, *N*-nitroso compound; NO_x_, total nitrate and nitrite; OTU, operational taxonomic unit; PCR, polymerase chain reaction; PMA, platelet-monocyte aggregate; XOR, xanthine oxidoreductase.
